# Ultrasmooth Micromilling of Stainless Steel by Ultrashort Pulsed Laser Ablation Using MHz Bursts

**DOI:** 10.1021/acsami.4c19517

**Published:** 2025-01-22

**Authors:** Xiao Jia, Folkert Vrijburg, Wei Zhang, Max Groenendijk, Yutao Pei

**Affiliations:** † Department of Advanced Production Engineering, Engineering and Technology Institute Groningen, Faculty of Science and Engineering, University of Groningen, Nijenborgh 4, Groningen, 9747 AG, The Netherlands; ‡ Philips Personal Care, Oliemolenstraat 5, Drachten, 9203 ZN, The Netherlands; § Lightmotif B.V., Pantheon 12, Enschede, 7521 PR, The Netherlands

**Keywords:** ultrashort pulsed laser, laser burst ablation, surface structures, ultrasmooth surface, stainless steel

## Abstract

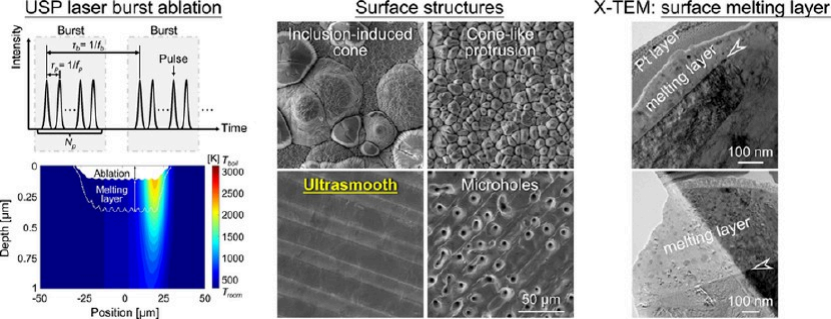

Ultrashort pulsed (USP) laser burst ablation has attracted numerous interests for its great potential in enhancing ablation efficiency and reducing the heat-affected zone. However, little attention has been paid to the influence of burst ablation on the processed surface quality. To fill this research gap, the present study conducts a comprehensive investigation on the surface processing of stainless steel using ultrashort pulsed laser burst ablation. Systematic experiments have been carried out to investigate influences of pulse number per burst (PpB), pulse fluence, and burst overlap ratio on surface quality and ablation efficiency. A two-dimensional model has been developed to unveil the fundamental thermodynamic process and evolution of ablation and melting in material during USP laser burst ablation. Compared to single-pulse ablation, the optimum ablation efficiency decreases with increasing PpB by less than 30% in burst ablation. Despite reduced ablation efficiency, burst-mode ablation can generate much better surface quality, achieving an ultrasmooth surface with an *S*_*a*_ roughness as low as 0.13 μm. Burst ablation generates distinctive surface structures compared to single-pulse ablation, and their formation mechanisms are scrutinized. The thickness of the surface melting layer is unveiled to determine surface morphology. Based on transmission electron microscopy (TEM) analysis and numerical simulation, a melting layer thickness between 100 and 320 nm is found to result in smooth surfaces. This work highlights the advantage of burst-mode ablation in achieving ultrasmooth surfaces on stainless steel and unveils the fundamental mechanisms of surface structures formation in USP laser burst ablation.

## 1 Introduction

Ultrashort pulsed (USP) lasers have been extensively employed for high-precision machining thanks to their ability to minimize thermal damages, which ensures a clean machining process without severe surface debris, recasting, melting and heat-affected zone (HAZ).^1,2^ The absence of thermal damage enables creation of high-precision surface structures spanning tens of nanometers to micrometers in size, including nanoholes,^3,4^ laser-induced periodic surface structure (LIPSS),^4,5^ cone-like protrusions,^6,7^ and inclusion induced cones.^8^ These multiscale surface structures offer diverse functionalities, such as coloring,^9,10^ self-cleaning,^11,12^ antireflection^13,14^ and friction reduction.^15^ However, their presence can compromise surface quality by increasing the surface roughness, making ultrashort pulsed laser processing unsuitable for applications requiring highly smooth surfaces, such as tools and mold fabrication.

To enhance surface quality in ultrashort pulsed laser processing, various methods have been proposed. The use of double-pulse ablation has shown promise in improving surface quality by precise manipulation of pulse energy and pulse interval time.^16,17^ Circularly polarized laser beams have been suggested as an alternative to linearly polarized beams to mitigate surface structures with fixed directions parallel or perpendicular to the polarization direction.^18^ A dual-process machining approach has been proposed to reduce the surface roughness by adding a fine polishing process after the primary laser ablation.^19^ Laser ablation in liquid (LAL) has proven effective in enhancing surface quality by constraining plasma expansion and suspending redeposited material.^20^ In LAL, electric/magnetic^21^ and ultrasonic fields^22^ have been introduced to enhance surface quality by controlling plasma ejection dynamics. Although these methods can reduce the surface roughness considerably, there are major drawbacks, such as complex experimental setup, prolonged processing time, and inability to completely eliminate the self-organized structures.

Ultrashort pulsed laser burst ablation has emerged as a promising solution for achieving high-efficiency and high-quality material processing with reduced HAZ.^23,24^ In the past years, while numerous studies have been devoted to improving ablation efficiency via burst ablation,^23−29^ only a few recent investigations explored the impact of burst ablation on surface quality.^25,30^ It has been unveiled that burst ablation works in a similar manner to long-pulse lasers,^25^ generating thick molten layer on the surface due to additional thermal effects. This surface molten layer has the ability to cover and eliminate rough surface features.^30^ However, the additional thermal effects introduced by burst ablation also have the potential to increase the heat-affected zone (HAZ). Moreover, using burst ablation, the surface molten layer can be created in a well-controlled way via proper selection of burst parameters so that it will not sacrifice the machining precision. So far, the influences of burst ablation parameters on the processed surface quality have not been systematically studied, and the formation mechanisms of surface structures in laser burst ablation have not been fully understood.

This study aims to understand the influences of ultrashort pulsed laser burst ablation on surface processing (ablation efficiency, surface quality, morphology, and microstructure change) of stainless steel and explore the feasibility of creating ultrasmooth surfaces using burst ablation. Systematic experiments are carried out to study the influences of pulse number per burst (PpB), pulse fluence, and burst overlap ratio on ablation efficiency and surface quality. A two-dimensional numerical model is developed to study the ablation process and melting layer formation during burst ablation and assist in understanding the formation mechanisms of surface structures.

## 2 Methods

### 2.1 Experimental Setup

AISI 301 stainless steel sheets with a thickness of 0.55 mm were used for the laser burst ablation process. The initial surface roughness of the specimen is 0.21 ± 0.02 μm, and the chemical compositions are provided in Table 1.

**Table 1:** Chemical Composition of AISI 301 Stainless Steel

Element	C	Si	Mn	P	S	Cr	Ni	Mo	N
Composition (%)	0.09	1.15	1.21	0.026	0.001	16.0–19.0	6.0–9.50	0.80	0.10

**Figure 1:**
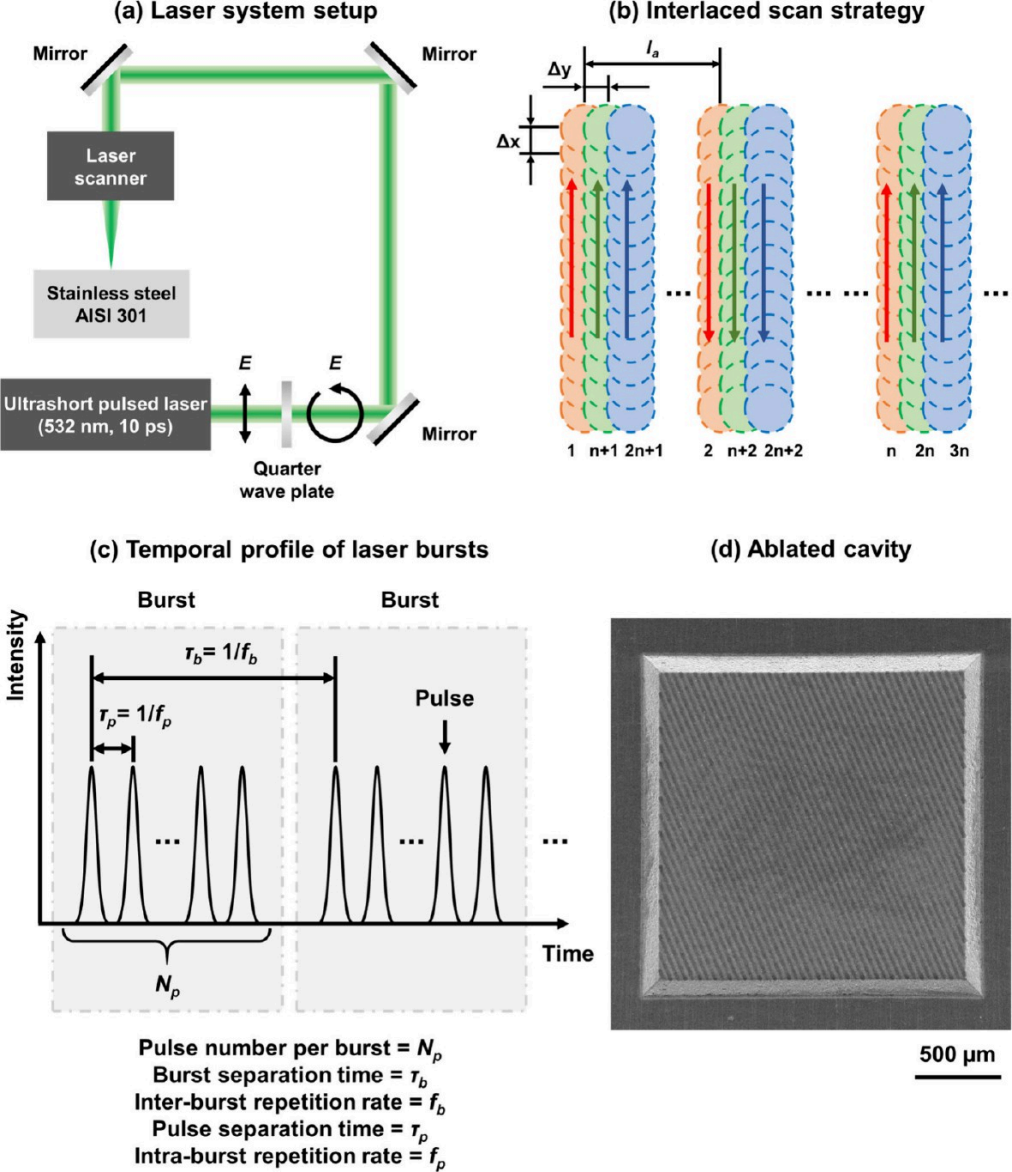
Schematic diagram of (a) laser system setup, (b) interlaced scan strategy, (c) temporal profile of laser bursts, and (d) ablated cavity. In (a), *E* represents the electric field of the laser beam. In (b), Δ*x*, Δ*y* and *l*_*a*_ represent the interburst distance, hatch distance, and interlacing distance, respectively. The pulse-to-pulse distance within a burst is much smaller than Δ*x* and is not shown in (b) for clarity.

Figure 1a depicts the laser system setup used for this study. A 23 W ultrashort pulsed laser from Lumentum (Picoblade 2, 10 ps, 532 nm, 82 MHz) was employed for ablation processing. The linearly polarized output laser beam was circularly polarized by a quarter-wave plate. A galvo scanner (Scanlab, intelliSCAN 10) with a maximum scan speed of 8.0 m/s was used for laser beam scanning. The laser beam (Gaussian shape, M^2^ < 1.2) was focused by an F-theta lens (Sill Optics) with a focal length of 100 mm, and the focal spot diameter (1/*e*^2^) was measured as 19.0 μm using a beam profiler (Primes MicroSpotMonitor).

The PpB was adjusted from 1 to 6, and the pulse fluence (peak fluence) was varied from 0.2 to 1.8 J/cm^2^. The burst overlap ratio was adjusted between 0.2 and 0.99 by changing the scanning speed, while the overlap ratio between scanning lines was fixed at a hatch distance of 5 μm. During laser scanning, an interlaced scanning strategy^31^ was utilized to improve the surface quality by extending the surface cooling time between scanning lines, as shown in Figure 1b. This scanning strategy is different from the conventional sequential scanning method in that an interlacing distance (*l*_*a*_) equivalent to a multiplication of the hatch distance was applied between successive scanning lines. Multiple laser passes are needed to complete the scanning layer. In this study, the interlacing value was set to 8 (*l*_*a*_ = 40 μm) and 8 laser passes are required to scan an entire layer. Sky-writing was carried out to ensure a constant scan speed in the processed area. In this mode, the acceleration and deceleration of scanning were performed outside of the processed area. To minimize periodic patterns due to repetitive laser scanning in the same direction, the scanning direction in each layer was rotated by a certain angle, which was determined in the following manner. For the first four layers, the scanning direction was rotated 90° with respect to the preceding layer. Starting from the fifth layer, a cyclic sequence of rotation angles (92°, 93°, 91.5°, and 92.5°) was adopted for every four consecutive layers. Figure 1c demonstrates the temporal profile of pulses in burst-mode laser ablation. The intraburst and interburst repetition rates were fixed at 82 MHz and 500 kHz, respectively. The experimental settings of the laser processing parameters are summarized in Table 2.

**Table 2:** Experimental Settings of USP Laser Scanning

Laser wavelength λ [nm]	532
Pulse duration τ [ps]	10
Focal spot radius ω_0_ [μm]	9.5
Hatch distance Δ*y* [μm]	5
Interlace distance *l*_*a*_ [μm]	40
Burst overlap ratio	0.20–0.99
PpB *N*_*p*_	1–6
Pulse fluence *F*_*p*_ [J/cm^2^]	0.2–1.8
Burst repetition rate *f*_*b*_ [kHz]	500
Intraburst pulse repetition rate *f*_*p*_ [MHz]	82

To investigate the influences of different laser parameters on ablation efficiency and surface quality, square cavities (2.0 × 2.0 mm^2^) with a depth of 0.1 mm (unless otherwise stated) were generated by laser ablation, as shown in Figure 1d. Ablation efficiency is defined as the ablation volume per burst energy, which is calculated as follows,^25,32^
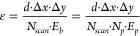
1where *d* is the cavity depth, Δ*x* is the interburst distance, Δ*y* is the hatch distance, *N*_*scan*_ is the layer number of laser scan, *N*_*p*_ is the PpB, and *E*_*b*_ and *E*_*p*_ are the burst energy and pulse energy, respectively.

A laser confocal microscope (Olympus LEXT 4100) was employed to measure the cavity depth and surface roughness. To eliminate systematic errors, for each laser ablation condition, two cavities were generated for the measurements. Based on the measured cavity depth, eq 1 was employed to calculate the ablation efficiency. In surface roughness measurements, the *S*_*a*_ value was determined on a 500 × 500 μm^2^ area. Within each cavity, three measurements were performed in different regions to obtain the average roughness. A Gaussian filter with a cutoff distance of 100 μm was applied in the measurements.

A scanning electron microscope (SEM, Tescan Lyra) was used to evaluate the surface morphology after laser ablation processing. Transmission electron microscopy (TEM) analysis was conducted to characterize the cross sections of surface layers. For TEM specimen preparation, C and Pt protective layers were sequentially deposited on the sample surface prior to ion milling. Then, a series of cross-sectional lamellae (approximately 1 μm thick) were created using the “lift-out procedure” facilitated by an FEI Helios G4 CX dual-beam microscope. These lamellae were moved over to a TEM grid made of copper and further thinned by a focused ion beam to a thickness between 50 and 100 nm. Bright-field TEM imaging was carried out using a JEOL-2200FS microscope with an acceleration voltage of 200 kV.

### 2.2 Numerical Simulation

**Figure 2:**
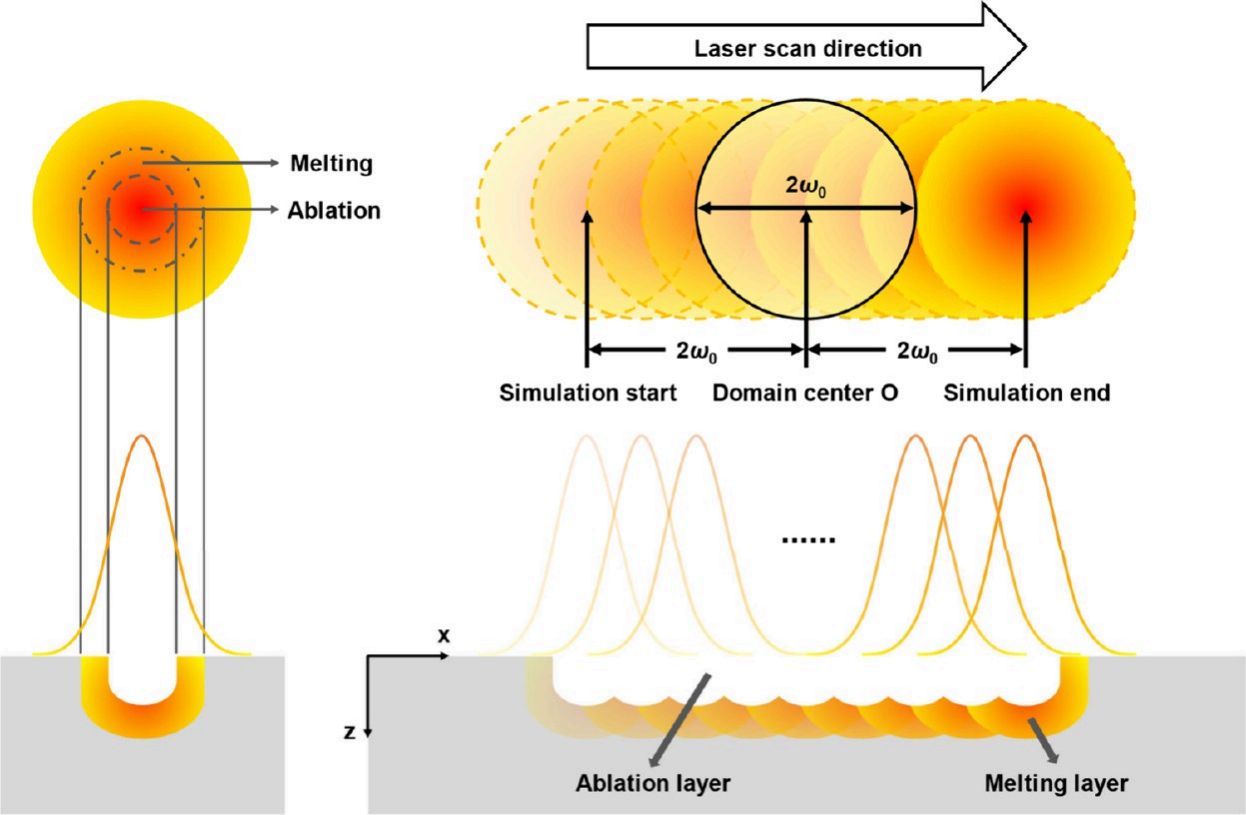
Schematic diagram of the ultrashort-pulsed laser burst ablation in the simulation. The different time intervals between bursts and pulses within a burst are not presented in the spatial profile of laser pulses.

During the laser milling process, the material undergoes ablation and melting, which are crucial to the formation of a surface morphology. To better understand the ablation process and formation of the melting layer during laser scanning, a two-dimensional model was developed to describe the temperature evolution along the centerline of the laser scanning path. The schematic diagram of this model is displayed in Figure 2. Details of this model can be found in the Supporting Information.

## 3 Results and Discussion

### 3.1 Effects of Pulse Fluence and Pulse Number Per Burst

**Figure 3:**
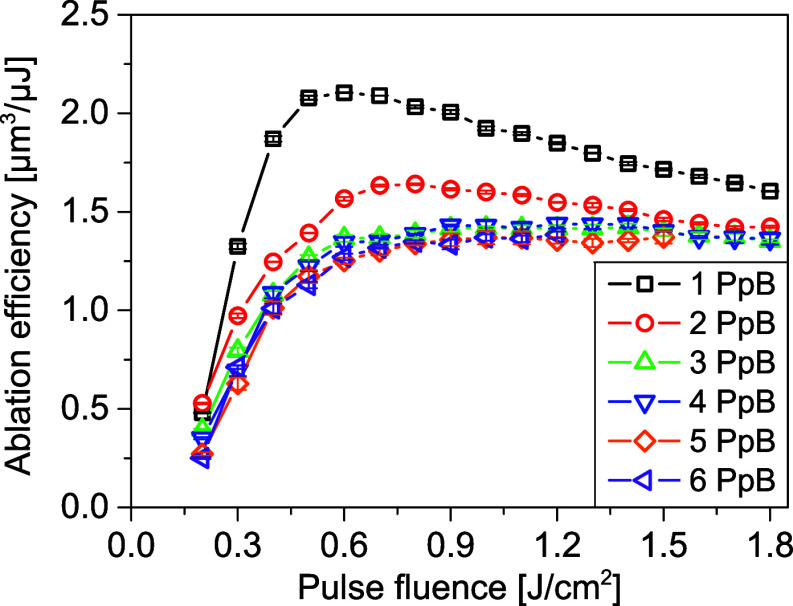
Ablation efficiency as a function of pulse fluence for different PpB. The burst overlap ratio is fixed at 0.9.

Ablation efficiency is an important factor to characterize the laser ablation process, which is defined as the ablation volume per unit of laser energy. Figure 3 displays the measured ablation efficiency at varying laser pulse fluences with different PpB. The burst overlap ratio is fixed at 0.9.

With increasing pulse fluences, for 1PpB, the ablation efficiency increases rapidly, reaching the optimum efficiency ∼2.0 μm^3^/μJ at 0.6 J/cm^2^ (optimum pulse fluence), and then gradually decreases. The optimum ablation efficiency agrees well with the results reported using USP lasers with comparable pulse duration.^33^ Reduction of ablation efficiency in low and high fluence regimes is caused by two different mechanisms. At pulse fluences below 0.6 J/cm^2^, the reduced ablation efficiency is mainly caused by low laser absorption rate due to weak electron excitation. At pulse fluences over 0.6 J/cm^2^, although electron excitation is strong enough to enhance laser absorption, high fluences can cause material overheating to temperatures much higher than the ablation temperature. The laser energy used for overheating is carried away by the ablated material, instead of being used for ablating more material,^34^ and the resultant ablation efficiency is thus reduced. The trend of the ablation efficiency with 2 PpB is similar to that of 1 PpB, while the optimum pulse fluence is slightly raised to 0.7 J/cm^2^. For 3–6 PpB, the ablation efficiency keeps increasing and saturates, without observing the decreasing trend, which is mainly resulted from the increased optimum fluence with increasing PpB.^24^

With increasing PpB, the optimum ablation efficiency decreases from ∼2.0 μm^3^/μJ (1 PpB) to ∼1.4 μm^3^/μJ (3–6 PpB). A similar phenomenon has been reported using MHz to GHz bursts for stainless steel^26,30^ and some other materials.^25−29^ This indicates that laser burst ablation cannot improve the ablation efficiency in a milling process as in the percussion drilling process.^23,35^ Plasma shielding^36−39^ has been widely hypothesized as the main reason for efficiency reduction, and this effect is dependent on the ablated material. For instance, in burst ablation of copper, plasma shielding has been reported to cause significantly reduced efficiency for 2 PpB, but the efficiency for 3 PpB is much higher than 2 PpB,^29^ or even higher than 1 PpB.^27^ However, this phenomenon is not observed in Figure 3 and the ablation efficiency monotonically decreases with increasing PpB. One possible explanation is that the stainless steel plasma is significantly stronger and has a lifetime much longer than copper plasma, so that the plasma shielding effect is still strong when the third pulse arrives. A further study on the plasma dynamics of stainless steel will be carried out to better understand this phenomenon.

**Figure 4:**
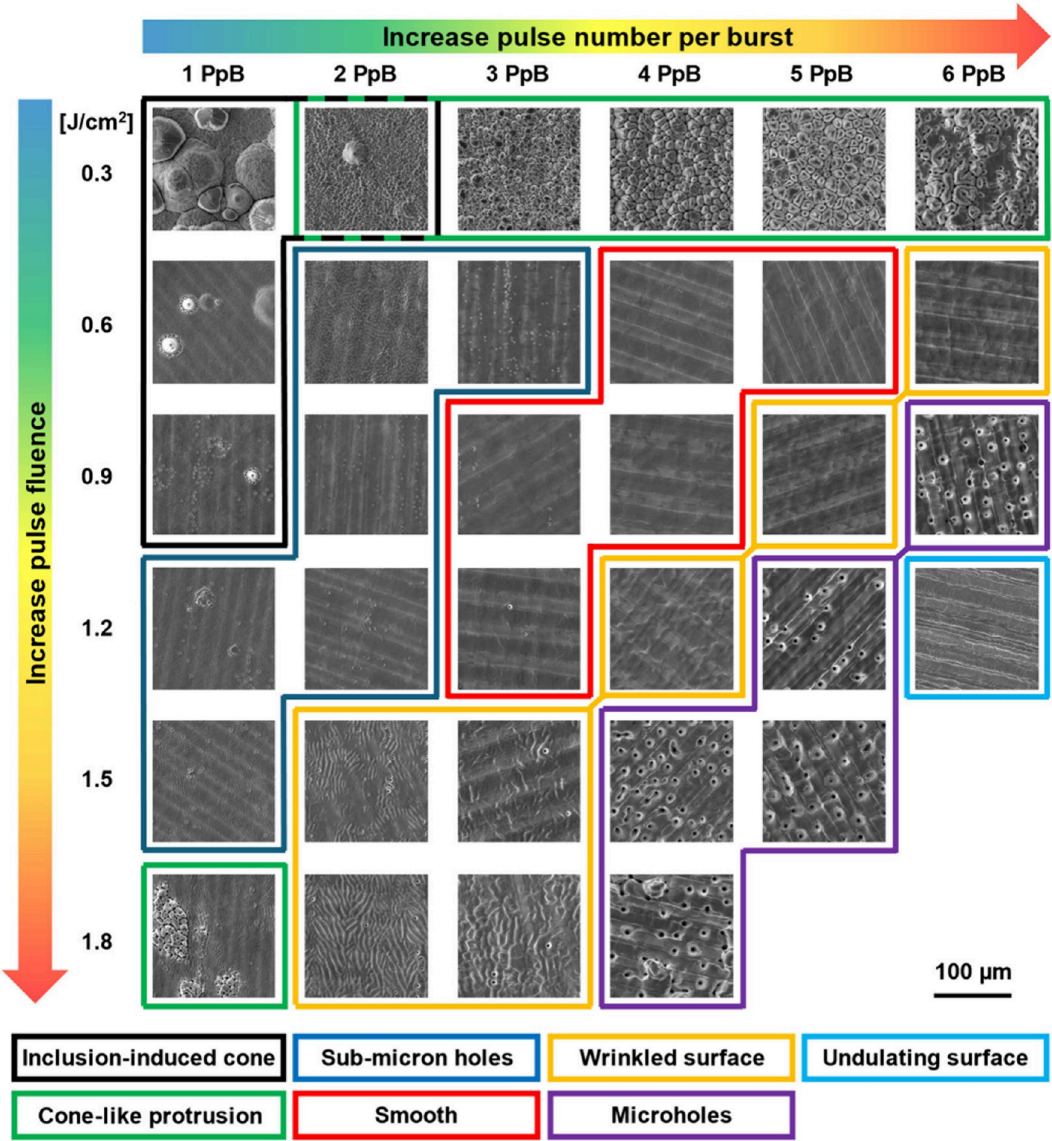
Laser-processed surface morphology at varying pulse fluences and PpB. The burst overlap ratio is fixed at 0.9. Different surface structures are marked in colored frames according to the structure types displayed at the bottom.

The surface morphology with different pulse fluences and PpB is displayed in Figure 4, and seven types of structures can be defined based on their characteristics. With increasing burst fluence, laser ablation becomes more thermal and surface structures change in the following sequence: *inclusion-induced cone* (IIC), *cone-like protrusion* (CLP), *submicron holes*, *smooth surface*, *wrinkled surface*, *microholes* and *undulating surface*.

IIC appears at the lowest burst fluence, for instance, with 1 PpB at 0.3–0.9 J/cm^2^. IIC formation is mainly caused by inclusions that have much higher ablation threshold than steel.^8^ Inclusions shield ablation of the material underneath and lead to cone formation. Early works^6,40^ suggest that inclusions are caused by ablation debris redeposition and oxidation. However, inclusions are recently found to be caused by impurities,^8^ which are mainly aluminum and magnesium oxide formed during stainless steel production. With increasing pulse fluences, the density of IIC decreases significantly since the ablation rate of inclusions increases, and inclusions can be efficiently removed before cone formation.

The second type of surface structures, CLP, occurs for 2–6 PpB at 0.3 J/cm^2^ and 1 PpB at 1.8 J/cm^2^. As reported in previous studies,^8^ CLP typically appears at high fluences for 1 PpB ablation, while the results in Figure 4 suggest that CLP can be generated at much lower pulse fluence for burst ablation. The dominating mechanism for CLP formation is preferential valley ablation (PVA).^6^ PVA occurs following the formation of rough precursor structures with higher ablation rates in the valleys. This is mainly because of higher laser fluences localized in the valley for two reasons. On one hand, the laser light reflected from the inclined surrounding area travels into the valleys and increases the local fluence.^41^ On the other hand, the valley area is usually smaller than the inclined surrounding areas, and this leads to a higher local fluence in the valley.

The third type *submicron holes* start to appear with 2 PpB and 0.6 J/cm^2^. The hole diameter is close to the laser wavelength, and the hole density decreases with increasing burst fluences. At higher burst fluences, when submicron holes disappear, for instance with 4 PpB and 0.6 J/cm^2^, the surface becomes very smooth. A hypothesis for the formation of smooth surface is that increasing melting layer thickness can fill in the holes or the gaps between CLPs and generate a flat surface.^42,43^ This hypothesis is consistent with the transition from submicron holes or CLPs to smooth surfaces; however, the fundamental process remains unclear and will be discussed in Section 3.3.

When the burst fluence is further increased, the smooth surface becomes wrinkled. This change is mainly attributed to the formation of an overthick melting layer that cannot flatten before solidification. With an even higher burst fluence, the surface starts to be covered by microholes with a diameter of ∼5 μm. At a further increased burst fluence, the melting layer becomes thick enough to prevent the formation of microholes. In this case, the thick molten material causes waviness between neighboring scanning tracks and the periodic structure caused by overlapped laser bursts can also be observed in the scanning tracks. It should be noted that the submicron holes and microholes in Figure 4 are different from the holes that coexist with cone structures.^6^ The two types of holes found in this work are generated on relatively smooth surfaces without cone formation.

It can also be observed that the surface structure is not solely determined by the burst fluence; rather, the same burst fluence with different combinations of PpB and pulse fluences can generate distinct surface structures. For instance, at a burst fluence of 7.2 J/cm^2^, microholes are generated for 4 PpB at 1.8 J/cm^2^, whereas an undulating surface is formed for 6 PpB at 1.2 J/cm^2^. Similarly, at a burst fluence of 2.4 J/cm^2^, submicrometer holes and smooth surfaces are formed for 2 and 4 PpB, respectively. This indicates a more pronounced thermal ablation process in laser bursts with higher PpB, which can generate thicker melting layers. The primary reason is the occurrence of strong thermal accumulation that becomes more prominent with increasing PpB,^24^ which contributes to thicker melting layers and results in different surface structures.

**Figure 5:**
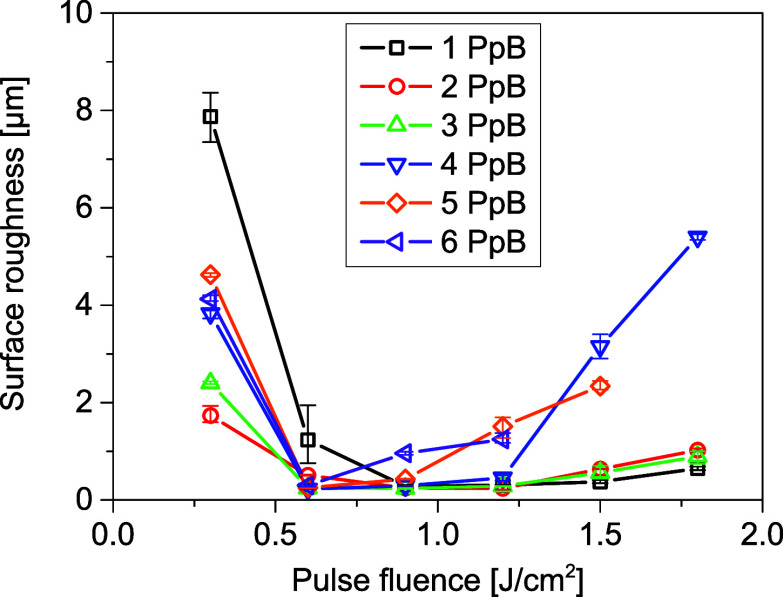
Surface roughness as a function of pulse fluence for different PpB. The burst overlap ratio is fixed at 0.9.

The surface roughness corresponding to different morphologies was measured and summarized in Figure 5. A roughness as low as ∼0.25 μm can be achieved on smooth surfaces. With increasing pulse fluences, the surface roughness first decreases and then reincreases. This variation trend is similar for different PpB, but the processing windows for low roughness are different. Generally, with increasing PpB, the processing window becomes narrower and moves to lower fluences. This window starts from 0.9 J/cm^2^ for 1PpB, while it starts from 0.6 J/cm^2^ for 2–6 PpB. At lower pulse fluences, IICs and CLPs are generated for 1 and 2–6 PpB, respectively. With increasing fluences, for 1–3 PpB, the roughness is stable for fluences below 1.2 J/cm^2^ and increases slowly from 1.2 to 1.8 J/cm^2^. For 4–6 PpB, a rapid increase of roughness can be observed, starting from 1.5, 1.2, and 0.9 J/cm^2^, respectively.

### 3.2 Effects of Burst Overlap Ratio

**Figure 6:**
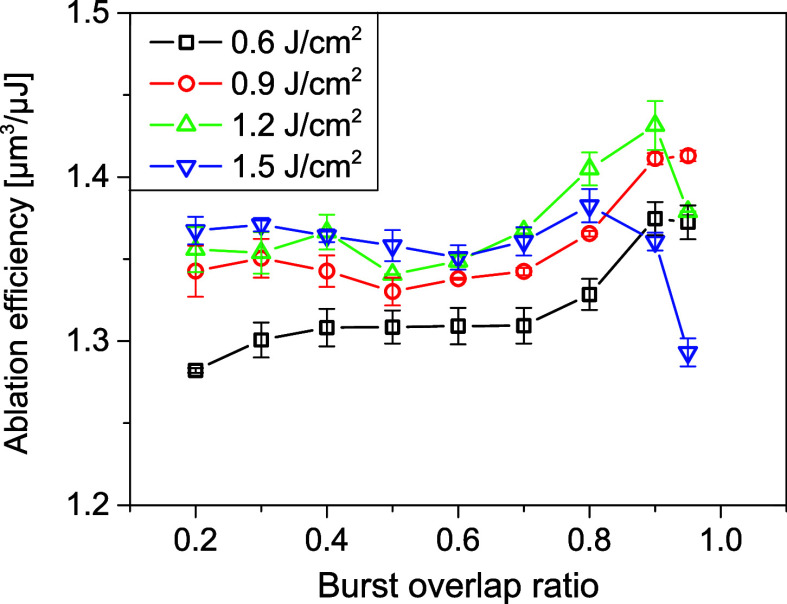
Ablation efficiency as a function of burst overlap ratio for different pulse fluences with 4 PpB.

Burst overlap ratio is another crucial parameter in laser ablation scanning. To understand the influences of the burst overlap ratio on ablation efficiency, surface morphology, and roughness, experiments were carried out for different burst overlap ratios and pulse fluences with 4 PpB. Figure 6 displays the measured ablation efficiency at varying burst overlap ratios at four pulses fluences. For 0.6 J/cm^2^, the ablation efficiency decreases with a reduction in the burst overlap ratio, and the decrease is less than 10% when the overlap ratio is reduced from 0.9 to 0.2. For higher fluences, this decreasing trend is ever less obvious, indicating insignificant influence of the burst overlap ratio on the ablation efficiency. An interesting phenomenon is observed that for high fluences of 1.2 and 1.5 J/cm^2^, when the overlap ratio is raised above a critical value (0.9 for 1.2 J/cm^2^ and 0.8 for 1.5 J/cm^2^), the ablation efficiency drops dramatically, and this reduction is more significant for higher fluences. This phenomenon is mainly caused by the formation of *chaotic* structures, as will be discussed later in this section.

**Figure 7:**
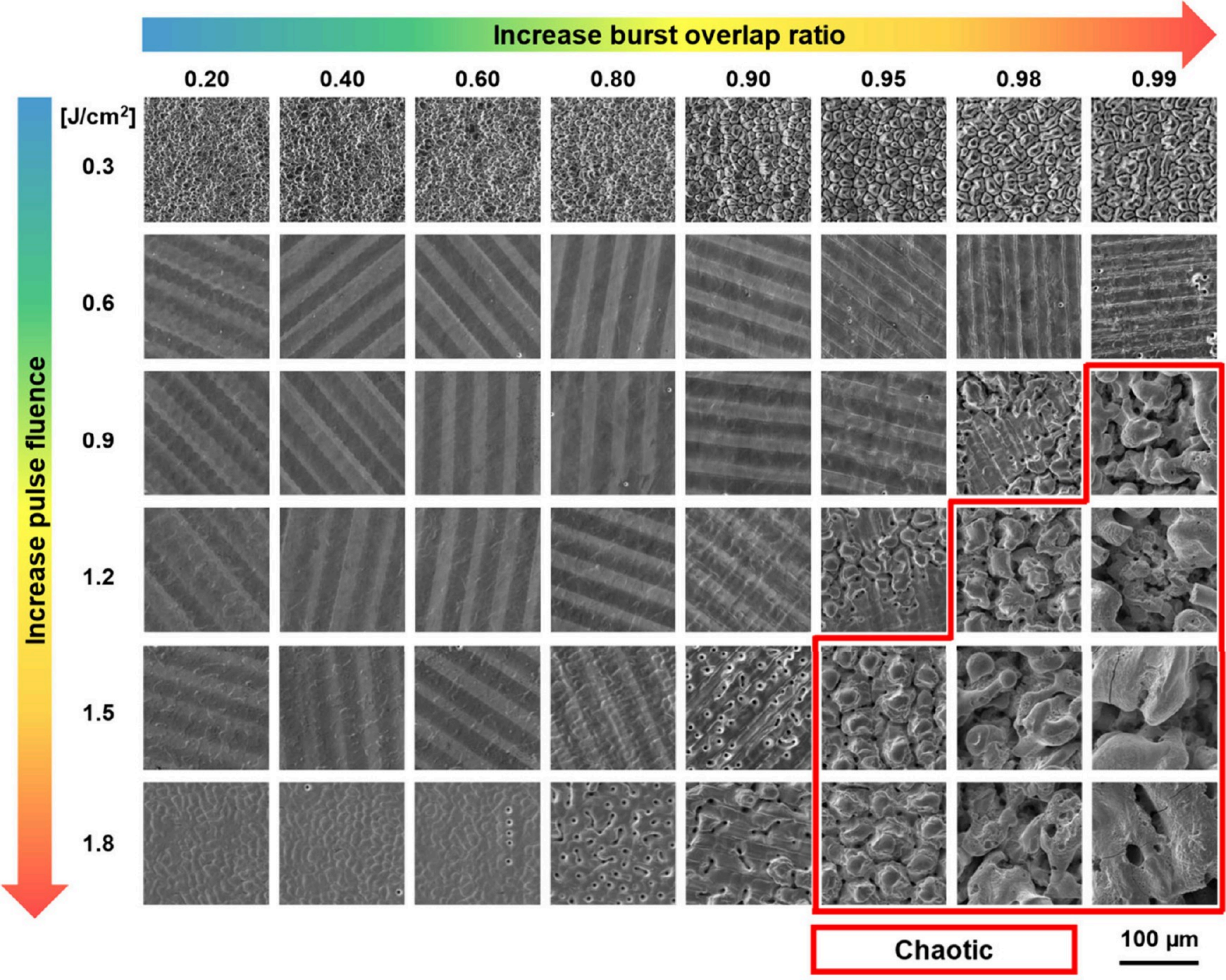
Laser-processed surface morphology at varying burst overlap ratios and pulse fluences. All experiments are performed with 4PpB. Chaotic structures are marked in a red frame.

The surface morphology variations with a burst overlap ratio at different pulse fluences are demonstrated in Figure 7. Most of the surface morphologies in Figure 7 have been observed in Figure 4, while the surface structures for a high burst overlap ratio at 0.9–1.8 J/cm^2^ are much rougher and not observed in Figure 4. This type of structure can be categorized as a *chaotic structures*. As has been mentioned, the generation of chaotic structures significantly reduces the ablation efficiency. This is because, on one hand, the laser light is significantly deflected and scattered by these chaotic structures, which reduces the local fluence. On the other hand, the material ablated from the valleys cannot be efficiently removed but is trapped by the chaotic structures.

At different fluences, the surface becomes smoother with decreasing burst overlap ratios. At 0.3 J/cm^2^, surfaces covered by CLPs are observed for different burst overlap ratios, and the cone size and intercone distance become smaller with a lower burst overlap ratio. At higher fluences, smooth surfaces can be generated, and the transition burst overlap ratio from rough to smooth surfaces decreases with increasing pulse fluences from 0.95 at 0.6 J/cm^2^ to 0.8 at 1.2 J/cm^2^. A mixture of different surface structures can be observed during transition from chaotic to smooth surfaces. For instance, CLPs and wrinkled surfaces coexist on the surface at 1.2 J/cm^2^ and a 0.95 burst overlap ratio. Once smooth surfaces are formed, no obvious change in surface morphology can be observed with further reducing burst overlap ratio until 0.2.

**Figure 8:**
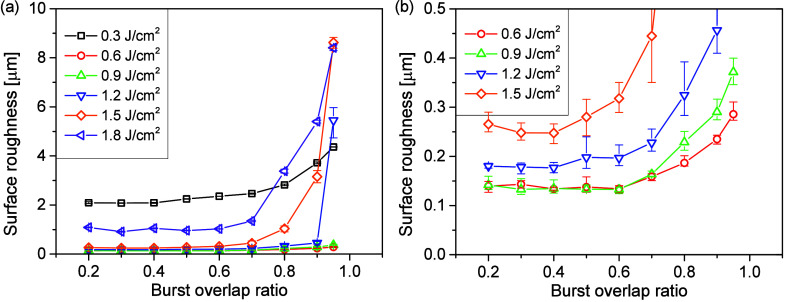
(a) Surface roughness as a function of burst overlap ratio for different pulse fluences and (b) zoom-in window for the low roughness region in (a). All experiments are performed with 4PpB.

The surface roughness corresponding to different cases in Figure 7 is displayed in Figure 8a. A decreasing trend of surface roughness with decreasing burst overlap ratios is demonstrated. While the lowest roughness for 0.3 J/cm^2^ and 1.8 J/cm^2^ is ∼2 μm and ∼1 μm, respectively, the roughness for other fluences can be much lower and a zoom-in window in the low roughness region is shown in Figure 8b. For 0.6, 0.9, and 1.2 J/cm^2^, no obvious change in the roughness can be observed when the burst overlap ratio is reduced below 0.6. For 1.5 J/cm^2^, the roughness keeps decreasing until 0.4 burst overlap ratio and reincreases slightly with further decreasing burst overlap ratios. The minimum roughness for 0.6 and 0.9 J/cm^2^ reaches as low as 0.13 μm, and for 1.2 and 1.5 J/cm^2^, the lowest roughnesses are 0.18 and 0.25 μm, respectively. Taken 0.3 μm as the roughness requirement for industrial applications, there is a wide processing window in burst overlap ratios for different pulse fluences and this processing window becomes smaller with increasing pulse fluences. At 0.6 J/cm^2^, the highest working burst overlap ratio is 0.95, while this is reduced to 0.5 when the pulse fluence is raised to 1.5 J/cm^2^.

In summary, high-quality (*S*_*a*_ of 0.13 μm) surfaces can be generated at high ablation efficiency (1.3–1.4 μm^3^/μJ) using 82 MHz USP burst-mode ablation. Table 3 compares the ablation efficiency and surface roughness of this work (1 and 4 PpB) with data from the literature. While the ablation efficiency of this work is comparable to previous studies for both single-pulse and burst mode ablation,^32,43−45^ the best surface quality achieved (*S*_*a*_ of 0.13 μm) surpasses that of earlier works. Notably, although the best surface quality (*S*_*a*_ of 0.12 μm) reported by Metzner et al.^30^ using 5 GHz laser bursts is comparable, the ablation efficiency in that study was significantly lower, particularly for burst-mode ablation (0.25 μm^3^/μJ for 4 PpB).

**Table 3:** Comparison of the Maximum Ablation Efficiency (ε) and Minimum Surface Roughness (*S*_*a*_) in Burst Ablation of Stainless Steel with Literature Data (τ-Pulse Duration, λ-Laser Wavelength, *f*_*p*_-Intra-burst Pulse Repetition Rate, and *f*_*b*_-Burst Repetition Rate)

		Laser parameters				Reference
ε [μm^3^/μJ]	*S*_*a*_ [μm]	τ [ps]	λ [nm]	*f*_*p*_ [MHz]	*f*_*b*_ [kHz]	
2.1 (1 PpB), 1.4 (4 PpB)	0.13	10	532	82	500	This work
2.25 (1 PpB), 1.58 (4 PpB)	N.A.	10	1064	82	200	[^32^]
1.5 (4 PpB, 0.5 J/cm^2^)	1.2	10	1030	65	50	[^43^]
1.25 (4 PpB, 1.5 J/cm^2^)	0.25					
2.67 (1 PpB), 1.67 (4 PpB)	N.A.	10	532	82	200	[^44^]
2.25 (1 PpB), 1.6 (4 PpB)	N.A.	10	1064	82	200	[^45^]
0.7 (5 PpB)	0.22	10	1030	62.5	200	[^46^]
0.25 (4 PpB)	0.12	10	1030	5000	N.A.	[^30^]

The superior surface quality achieved in this work can satisfy the requirements of many industrial applications without the need for postprocessing, saving numerous time, effort, and costs. The ablation efficiency can be further improved by reducing the pulse duration to subpicosecond regimes.^8^ A further study is necessary to compare the surface quality processed by femtosecond and picosecond lasers by using burst ablation.

### 3.3 Surface Structure Formation Mechanisms

#### 3.3.1 Surface Morphology Evolution

**Figure 9:**
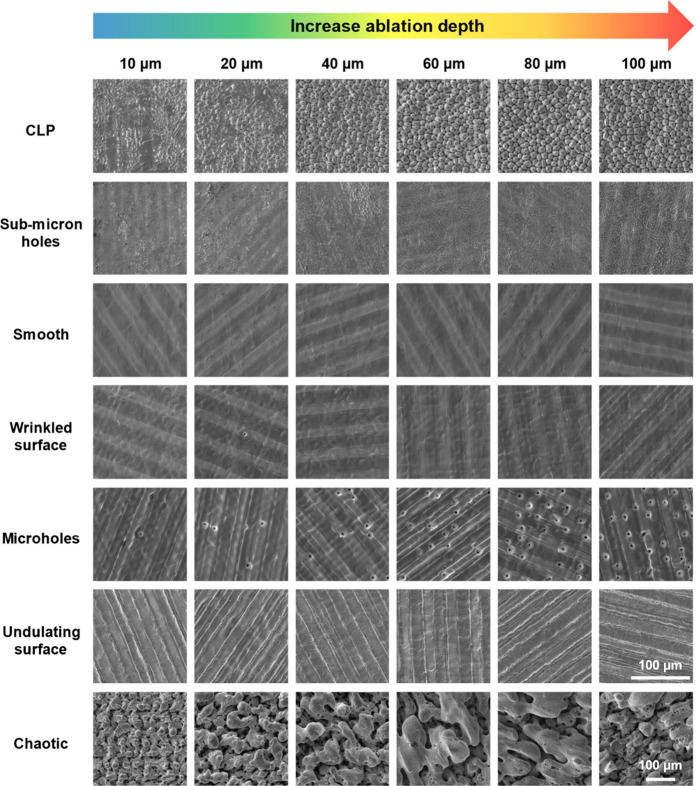
Evolution of surface structure morphology with an increasing ablation depth for different types of structures. The scale bar is the same for all the images except for the chaotic structures.

As mentioned in Section 3.1 and 3.2, eight types of surface structures can be generated by USP laser burst ablation. To understand the formation processes of these structures, the evolution of surface morphology with increasing ablation depths is shown in Figure 9. For CLP, submicrometer holes, microholes, and chaotic structures, the surface morphology changes mostly within 40 μm depth and becomes stable with deeper ablation. In contrast, there is no obvious change in surface morphology for smooth, wrinkled, and undulating surfaces at different ablation depths.

Preferential valley ablation (PVA)^6^ has been widely hypothesized as the dominating mechanism for the development of CLPs from precursor structures. These precursor structures can be formed in multiple ways, such as defects, nonuniform ablation and complex hydrodynamics.^47^ As shown in the top-left panel of Figure 9, at an ablation depth of 10 μm, the surface is already covered by grooves, pits, and holes, which serve as precursor structures for CLPs formation. With increasing ablation depth, these precursor structures become more regular cones, and there is no change in the cone size.

Full coverage of submicron holes on the ablated surface has not been reported before, and the existence of inclusions is expected to cause hole formation. On one hand, these holes can be observed in the surrounding area of the inclusions, as seen on the surfaces ablated with 1 PpB and 0.6–1.5 J/cm^2^ (Figure 4). For 2 PpB and 0.6 J/cm^2^, although IIC does not form, since inclusions cannot be removed instantly, when laser light interacts with inclusions, hot spots can be generated around inclusions from enhanced electric fields by localized surface plasmon resonance with pulses in the laser burst,^4^ and these hot spots can lead to the formation of submicron holes. On the other hand, the distribution of submicron holes is random, which is consistent with the random distribution of inclusions. Once these holes are formed on the surface, they can persist with an increasing ablation depth. Since the inclusions are distributed at different depths, the holes will accumulate with increasing ablation depth, and their density increases, as can be seen in Figure 9.

Surface smoothening by melting material has been proposed as the mechanism for smooth surfaces formation in laser burst ablation.^43^ According to this hypothesis, laser bursts generate thick melting material that fills the gaps between CLPs or in the holes, resulting in smooth surfaces. This aligns with the observation that CLPs and submicrometer holes disappear with increasing melting layer thickness at higher burst fluences. However, the fundamental process remains elusive, particularly whether rough structures (such as CLPs) are formed before the surface is smoothened by the melting material. Based on Figure 9, it is evident that smooth surfaces can be generated from the beginning and rough structures never appear with ablation depth. This suggests that the melting material smoothens the surfaces by eliminating precursor structures rather than filling gaps between CLPs or in holes after their formation. This feature is highly beneficial for industrial applications, as there is no limit on the ablation depth for creating smooth surfaces.

**Figure 10:**
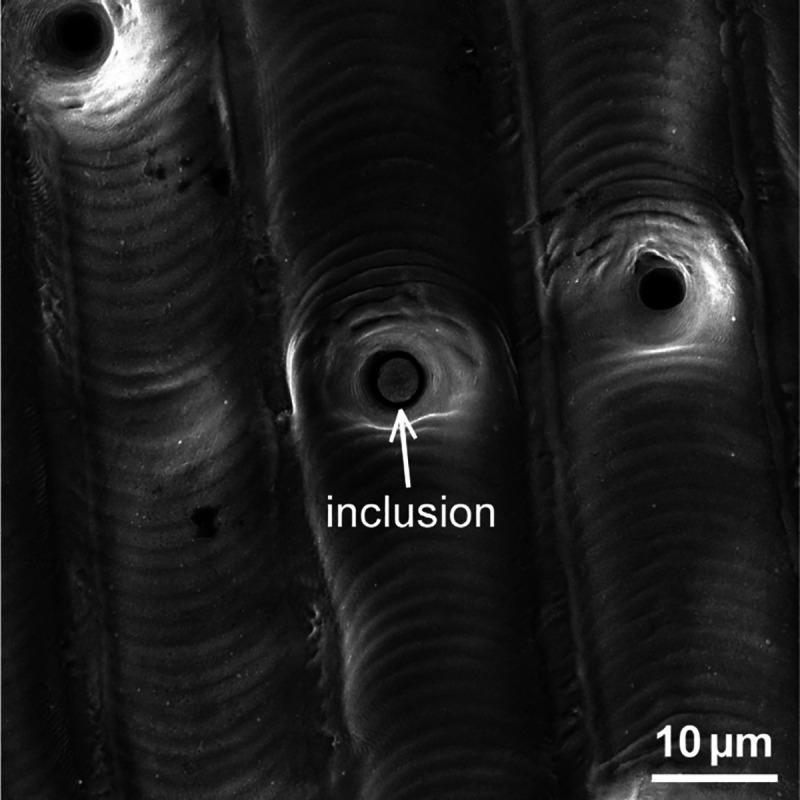
An incompletely ablated inclusion inside a microhole (the surface was fabricated with laser bursts of 6 PpB, 0.9 J/cm^2^ and 0.9 burst overlap ratio).

Further increase of burst fluence results in a thicker melting layer, giving rise to wrinkled surfaces, microholes, undulating surface, and chaotic surfaces in sequence. The occurrence of wrinkled surfaces can be understood by considering an excessively thick melting layer that cannot spread uniformly over the surface before cooling down. However, the appearance of microholes seems contradictory to the melting smoothening effect. It is likely that inclusions contribute to the formation of these microholes, since we observed a microhole with an incompletely ablated inclusion inside (see Figure 10). Additionally, an increasing density of microholes with increasing ablation depth can be observed in Figure 9, which indicates microhole accumulation similar to submicron holes. Meanwhile, the microholes are distributed randomly, which is consistent with the random distribution of inclusions. When inclusions are removed by laser ablation, a hole is left on the surface (Figure 9). This should also happen for smooth and wrinkled surfaces, where the melting material is expected to fill these holes. At the laser condition for forming microholes, the melting material is thicker, and these holes should be filled quickly. However, this is contrary to the generation of microholes. Therefore, there should be another mechanism competing with the melting smoothening effect. One plausible explanation is that when the burst fluence exceeds that for wrinkled surfaces, PVA becomes strong enough to compete with the melting smoothening effect. Once microholes are formed due to ablation of inclusions, PVA maintains these microholes and prevents the melting material from filling in and smoothing the surface.

Undulating surfaces are manifested when a thicker melting layer is generated that can fill in microholes. On the undulating surface, the melting material in the scanning tracks can remain steady and the overlap marks between bursts can be observed. At different ablation depths, the undulating surface does not change the morphology. However, when the melting material is even thicker, the material in the scanning tracks becomes unsteady, and the scanning tracks cannot maintain their shape. In such conditions, the hydrodynamics of the melting material becomes significant, leading to the formation of droplets on the surface through material ejection and redeposition. With increasing ablation depth, the surface structure becomes increasingly irregular and results in chaotic structures. The formation process of chaotic structures is similar to the above-surface growth structures reported by Zuhlke et al.,^7^ where the flow of surface melt and redeposition of ablated material are the dominating mechanisms. Due to intense material redeposition, the final surfaces of chaotic structures can be higher than the initial material surface, which results in significantly reduced ablation efficiency, as discussed in Section 3.2.

#### 3.3.2 Melting Layer Thickness

**Figure 11:**
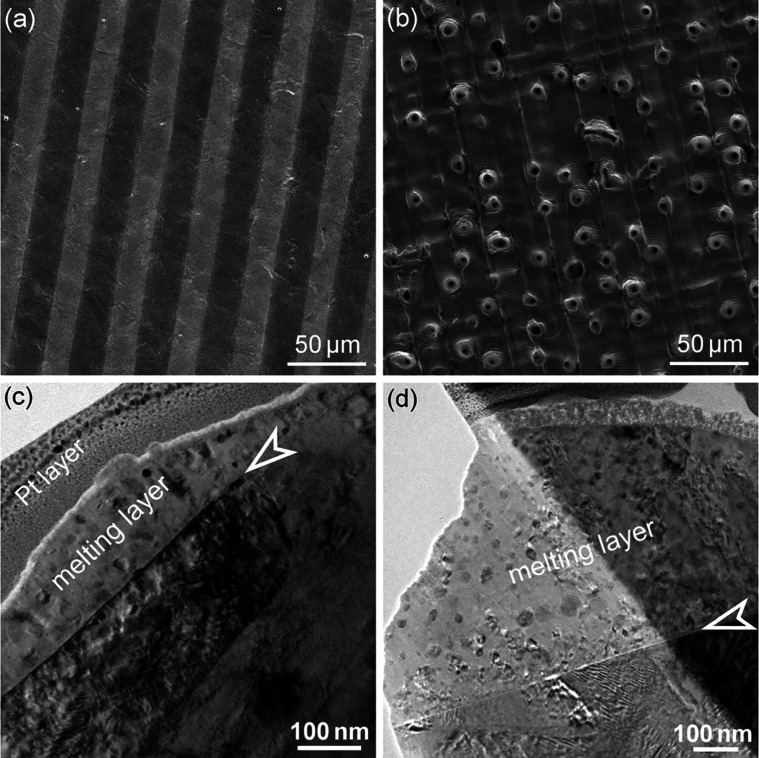
(a) SEM image of a smooth surface generated at 0.6 J/cm^2^, 4 PpB and 0.8 burst overlap ratio, and (b) a surface with microholes generated at 0.9 J/cm^2^, 6 PpB and 0.9 burst overlap ratio. (c,d) TEM images for the surface melting layers in (a) and (b), respectively.

In Section 3.3.1, it has been hypothesized that the melting layer thickness plays an important role in determining the surface morphology in laser burst ablation. To the best of the authors’ knowledge, no previous study has experimentally characterized the melting layer. To address this gap, TEM has been carried out to determine the melting layer thickness for the case of smooth surfaces and microholes, as shown in Figure 11.

As depicted in Figure 11c and 11d, there is a distinct interface between the surface material and the base material as indicated by an arrow. The material above this interface is identified as the melting layer formed by the USP laser burst ablation. It is revealed that for a smooth surface, the maximum thickness of melting layer is measured as ∼150 nm in Figure 11c. In contrast, when microholes are formed on the surface (Figure 11d), this melting layer becomes as thick as ∼570 nm. These TEM cross-sectional observations corroborate the hypothesis that the surface melting layer is responsible for smoothening the surface, and overthick melting layers result in nonsmooth surfaces, such as microholes.

**Figure 12:**
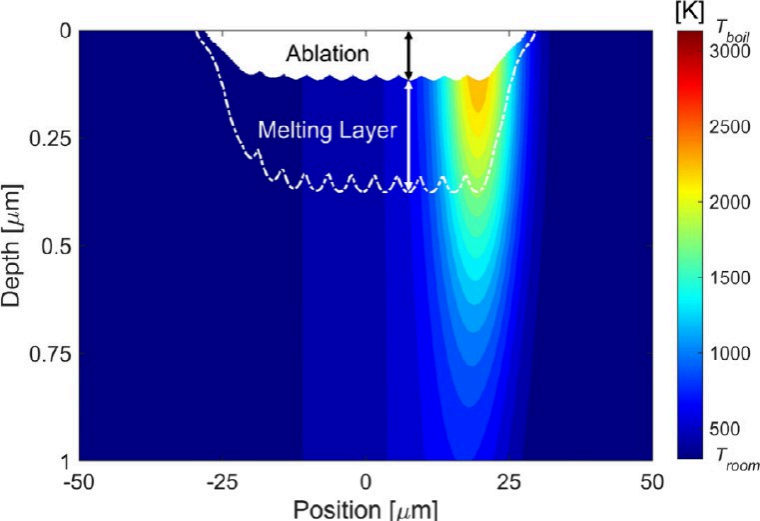
Temperature distribution at 12.2 ns after the last pulse in laser scanning for 4 PpB, 0.9 J/cm^2^ and 0.8 burst overlap ratio. The white part represents the ablated material, and the dash-dot line indicates the melting isotherm. The distance between the ablation bottom and the melting isotherm indicates the melting layer thickness.

To understand the melting layer formation process and evaluate the melting layer thickness corresponding to different morphologies, numerical simulations have been carried out in the same conditions as used for experiments. Figure 12 demonstrates the temperature distribution at 12.2 ns after the last pulse in laser scanning for 4 PpB, 0.9 J/cm^2^ and 0.8 burst overlap ratio. In Figure 12, the white area represents the ablated material, and the white dashed-dotted line indicates the melting isotherm. The simulation reveals that, due to burst-to-burst overlap, the ablation depth initially increases at the beginning of the ablation track, becomes steady, and finally decreases as laser beam scanning approaches the end of the track, as shown in the Supporting Information. The melting isotherm exhibits a similar profile. When both ablation and melting become steady, the distance between the ablation bottom and the melting isotherm is taken as the melting layer thickness.

**Figure 13:**
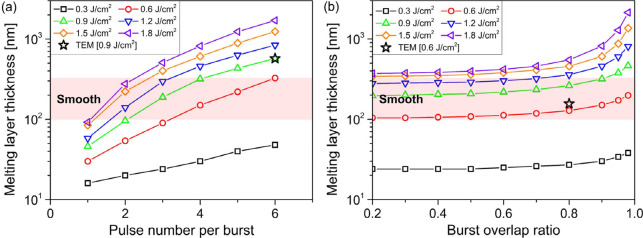
Simulation of melting layer thickness at different pulse fluences as a function of (a) PpB (0.9 burst overlap ratio) and (b) the burst overlap ratio (4 PpB). TEM measurements are marked in orange hollow stars. The range of melting layer thickness corresponding to smooth surfaces is marked in light red.

Figure 13 displays the calculated melting layer thickness at different laser burst conditions, and the simulation results agree with the TEM measurements. In Figure 13a, an increase in the melting layer thickness is observed with increasing PpB and pulse fluence, consistent with the morphological changes shown in Figure 4. This supports the hypothesis that the melting layer thickness is a crucial factor influencing surface morphology. In Figure 13b, it is found that the melting layer thickness decreases rapidly with a reducing burst overlap ratio and becomes stable when the burst overlap ratio falls below 0.8. This is in excellent agreement with the fact that surfaces become smoother as the burst overlap ratio decreases and the surface morphology remains almost unchanged with further decreasing burst overlap ratios (as shown in Figure 7). Based on Figures 4, 7 and 13, the correlation between melting layer thickness and surface morphology can be established. The melting layer thicknesses corresponding to different surface morphologies are listed in Table 4, and the thickness range for smooth surfaces is highlighted in Figure 13. It is found that, with a melting layer thickness ranging from 100 to 320 nm, highly smooth surfaces can be generated by laser burst ablation.

**Table 4:** Melting Layer Thickness Corresponds to Different Surface Morpholgies

Surface structures	Melting layer thickness [nm]
IIC and CLP	<50
Submicron holes	50–100
Smooth	100–320
Wrinkled	320–450
Microholes	450–700
Undulating surface	700–850
Chaotic	>850

## 4 Conclusions

In this study, laser burst ablation has been investigated on stainless steel to understand the influences of PpB (1–6), pulse fluence (0.2–1.8 J/cm^2^) and burst overlap ratio (0.20–0.99) on the ablation efficiency, surface roughness, and morphology. A two-dimensional model has been developed to unveil the ablation and melting process during USP laser beam scanning. It is found that the ablation efficiency is significantly influenced by pulse fluence and PpB. For 1 and 2 PpB, the ablation efficiency initially increases with pulse fluence and then decreases when the fluence exceeds the optimum fluence of 0.6 J/cm^2^. For 3–6 PpB, the ablation efficiency keeps increasing and saturates with increasing fluences, and the maximum ablation efficiency is 30% lower than that of 1 PpB. The influence of burst overlap ratio on ablation efficiency is much smaller. When the burst overlap ratio is too high (typically over 0.8–0.9), the ablation efficiency decreases sharply due to the formation of chaotic structures. Eight types of surface structures, namely, inclusion-induced cone (IIC), cone-like protrusion (CLP), submicron holes, smooth, wrinkled surface, microholes, undulating, and chaotic surface, can be generated by USP laser burst ablation. Highly smooth surfaces with *S*_*a*_ of 0.13 μm can be achieved at an ablation efficiency of 1.3–1.4 μm^3^/μJ. Two new surface structures are for the first time observed, namely, submicron holes and microholes, and they are mainly formed by inclusions. The surface melting layer thickness is found to be the dominating factor in determining surface morphology. The melting layer thickness was measured by TEM and calculated by the numerical model. The good correlation between the melting layer thickness and the surface structures has been demonstrated, and the melting layer thicknesses corresponding to different structures have been determined. A melting layer with a thickness between 100 and 320 nm can eliminate the formation of surface precursor structures and result in a highly smooth surface. A melting layer thinner than 100 nm corresponds to the formation of IIC, CLP and submicron holes on the surface, since these structures cannot be covered by the melting layer. In contrast, an overthick melting layer results in the formation of microholes, wrinkled, undulating, and chaotic surfaces due to more complicated hydrodynamics of the molten material. This study highlights the capability of USP laser burst ablation in creating ultrasmooth surfaces on stainless steel and improves the understanding of formation mechanisms for surface structures in burst ablation by ultrashort pulsed lasers.

## Data Availability

Data will be made available on request.

## Supporting Information Available

S1 Numerical simulation (PDF)
S2 A video to demonstrate the temperature distribution in stainless steel during ablation and melting layer formation in USP laser beam scanning with 4 PpB burst, 0.9 J/cm^2^ pulse fluence and 0.8 burst overlap ratio (MP4)

## References

[ref1] Mannion P. T.; Magee J.; Coyne E.; O’Connor G. M.; Glynn T. J. The Effect of Damage Accumulation Behaviour on Ablation Thresholds and Damage Morphology in Ultrafast Laser Micro-Machining of Common Metals in Air. Appl. Surf. Sci. 2004, 233 (1–4), 275–287. 10.1016/j.apsusc.2004.03.229.

[ref2] Nolte S.; Momma C.; Jacobs H.; Tünnermann A.; Chichkov B. N.; Wellegehausen B.; Welling H. Ablation of Metals by Ultrashort Laser Pulses. J. Opt. Soc. Am. B 1997, 14 (10), 2716–2722. 10.1364/JOSAB.14.002716.

[ref3] Zhang C.-Y.; Yao J.-W.; Liu H.-Y.; Dai Q.-F.; Wu L.-J.; Lan S.; Trofimov V. A.; Lysak T. M. Colorizing Silicon Surface with Regular Nanohole Arrays Induced by Femtosecond Laser Pulses. Opt. Lett. 2012, 37 (6), 1106–1108. 10.1364/OL.37.001106. 22446240

[ref4] Kawabata S.; Bai S.; Obata K.; Miyaji G.; Sugioka K. Two-Dimensional Laser-Induced Periodic Surface Structures Formed on Crystalline Silicon by GHz Burst Mode Femtosecond Laser Pulses. Int. J. Extrem. Manuf. 2023, 5 (1), 015004 10.1088/2631-7990/acb133.

[ref5] Huang M.; Zhao F.; Cheng Y.; Xu N.; Xu Z. Origin of Laser-Induced near-Subwavelength Ripples: Interference between Surface Plasmons and Incident Laser. ACS Nano 2009, 3 (12), 4062–4070. 10.1021/nn900654v. 20025303

[ref6] Ling E. J. Y.; Saïd J.; Brodusch N.; Gauvin R.; Servio P.; Kietzig A. M. Investigating and Understanding the Effects of Multiple Femtosecond Laser Scans on the Surface Topography of Stainless Steel 304 and Titanium. Appl. Surf. Sci. 2015, 353, 512–521. 10.1016/j.apsusc.2015.06.137.

[ref7] Zuhlke C. A.; Anderson T. P.; Alexander D. R. Formation of Multiscale Surface Structures on Nickel via above Surface Growth and below Surface Growth Mechanisms Using Femtosecond Laser Pulses. Opt. Express 2013, 21 (7), 8460–8473. 10.1364/OE.21.008460. 23571936

[ref8] Villerius V.; Kooiker H.; Post J.; Pei Y. T. Ultrashort Pulsed Laser Ablation of Stainless Steels. Int. J. Mach. Tools Manuf. 2019, 138, 27–35. 10.1016/j.ijmachtools.2018.11.003.

[ref9] Romano J. M.; Garcia-Giron A.; Penchev P.; Dimov S. Triangular Laser-Induced Submicron Textures for Functionalising Stainless Steel Surfaces. Appl. Surf. Sci. 2018, 440, 162–169. 10.1016/j.apsusc.2018.01.086.

[ref10] Geng J.; Xu L.; Yan W.; Shi L.; Qiu M. High-Speed Laser Writing of Structural Colors for Full-Color Inkless Printing. Nat. Commun. 2023 141 2023, 14 (1), 565 10.1038/s41467-023-36275-9. PMC9894925 36732539

[ref11] Sciancalepore C.; Gemini L.; Romoli L.; Bondioli F. Study of the Wettability Behavior of Stainless Steel Surfaces after Ultrafast Laser Texturing. Surf. Coat. Technol. 2018, 352, 370–377. 10.1016/j.surfcoat.2018.08.030.

[ref12] Milles S.; Soldera M.; Kuntze T.; Lasagni A. F. Characterization of Self-Cleaning Properties on Superhydrophobic Aluminum Surfaces Fabricated by Direct Laser Writing and Direct Laser Interference Patterning. Appl. Surf. Sci. 2020, 525, 146518 10.1016/j.apsusc.2020.146518.

[ref13] Fan P.; Bai B.; Zhong M.; Zhang H.; Long J.; Han J.; Wang W.; Jin G. General Strategy toward Dual-Scale-Controlled Metallic Micro-Nano Hybrid Structures with Ultralow Reflectance. ACS Nano 2017, 11 (7), 7401–7408. 10.1021/acsnano.7b03673. 28665579

[ref14] Vorobyev A. Y.; Guo C. Enhanced Absorptance of Gold Following Multipulse Femtosecond Laser Ablation. Phys. Rev. B 2005, 72 (19), 195422 10.1103/PhysRevB.72.195422.

[ref15] Samanta A.; Huang W.; Lee K.; He X.; Kumara C.; Qu J.; Ding H. Role of Surface Wetting on Tribological Behavior for Laser Nanotextured Steel Using Ionic Liquid Lubricants. J. Manuf. Process. 2023, 95, 302–311. 10.1016/j.jmapro.2023.04.031.

[ref16] Li T.; Guo Y.; Mizutani M.; Xu S. Surface Smoothing of Bulk Metallic Glasses by Femtosecond Laser Double-Pulse Irradiation. Surf. Coat. Technol. 2021, 408, 126803 10.1016/j.surfcoat.2020.126803.

[ref17] Schille J.; Schneider L.; Kraft S.; Hartwig L.; Loeschner U. Experimental Study on Double-Pulse Laser Ablation of Steel upon Multiple Parallel-Polarized Ultrashort-Pulse Irradiations. Appl. Phys. A: Mater. Sci. Process. 2016, 122 (7), 644 10.1007/s00339-016-0169-6.

[ref18] Durbach S.; Hampp N. Scan Direction of Circularly Polarized Laser Beam Determines the Orientation of Laser-Induced Periodic Surface Structures (LIPSSs) on Silicon. Appl. Phys. Lett. 2022, 121 (25), 251601 10.1063/5.0128227.

[ref19] Sassmannshausen A.; Brenner A.; Finger J. Ultrashort Pulse Laser Polishing by Continuous Surface Melting. J. Mater. Process. Technol. 2021, 293, 117058 10.1016/j.jmatprotec.2021.117058.

[ref20] Zhang D.; Sugioka K.; Zhang D.; Sugioka K. Hierarchical Microstructures with High Spatial Frequency Laser Induced Periodic Surface Structures Possessing Different Orientations Created by Femtosecond Laser Ablation of Silicon in Liquids. Opto-Electronic Adv. 2019, 2 (3), 19000201 10.29026/oea.2019.190002.

[ref21] Maksimovic J.; Ng S. H.; Katkus T.; An Le N. H.; Chon J. W. M.; Cowie B. C. C.; Yang T.; Bellouard Y.; Juodkazis S. Ablation in Externally Applied Electric and Magnetic Fields. Nanomaterials 2020, 10 (2), 182 10.3390/nano10020182. 31972998 PMC7074962

[ref22] Li P.; Liu B.; Li L.; Gong Y.; Zhou J.; Lu J. Study on Surface Quality of Ultrasonic Assisted Underwater Laser Polishing. J. Mater. Res. Technol. 2023, 27, 5761–5771. 10.1016/j.jmrt.2023.11.088.

[ref23] Kerse C.; Kalaycloĝlu H.; Elahi P.; Çetin B.; Kesim D. K.; Akçaalan Ö.; Yavaş S.; Aşlk M. D.; Öktem B.; Hoogland H.; Holzwarth R.; Ilday F. Ö. Ablation-Cooled Material Removal with Ultrafast Bursts of Pulses. Nature 2016, 537 (7618), 84–88. 10.1038/nature18619. 27409814

[ref24] Jia X.; Zhao X. Ultrafast Laser Ablation of Copper with GHz Bursts: A Numerical Study on the Effects of Repetition Rate, Pulse Number and Burst Fluence on Ablation Efficiency and Heat-Affected Zone. Opt. Laser Technol. 2023, 158, 108803 10.1016/j.optlastec.2022.108803.

[ref25] Domke M.; Matylitsky V.; Stroj S. Surface Ablation Efficiency and Quality of Fs Lasers in Single-Pulse Mode, Fs Lasers in Burst Mode, and Ns Lasers. Appl. Surf. Sci. 2020, 505, 144594 10.1016/j.apsusc.2019.144594.

[ref26] Žemaitis A.; Gaidys M.; Gečys P.; Barkauskas M.; Gedvilas M. Femtosecond Laser Ablation by Bibursts in the MHz and GHz Pulse Repetition Rates. Opt. Express 2021, 29 (5), 7641–7653. 10.1364/OE.417883. 33726261

[ref27] Žemaitis A.; Gečys P.; Barkauskas M.; Račiukaitis G.; Gedvilas M. Highly-Efficient Laser Ablation of Copper by Bursts of Ultrashort Tuneable (Fs-Ps) Pulses. Sci. Rep. 2019, 9 (1), 12280 10.1038/s41598-019-48779-w. 31439881 PMC6706424

[ref28] Bonamis G.; Audouard E.; Hönninger C.; Lopez J.; Mishchik K.; Mottay E.; Manek-Hönninger I. Systematic Study of Laser Ablation with GHz Bursts of Femtosecond Pulses. Opt. Express 2020, 28 (19), 27702–27714. 10.1364/OE.400624. 32988058

[ref29] Neuenschwander B.; Jaeggi B.; Foerster D. J.; Kramer T.; Remund S. Influence of the Burst Mode onto the Specific Removal Rate for Metals and Semiconductors. J. Laser Appl. 2019, 31 (2), 022203 10.2351/1.5096083.

[ref30] Metzner D.; Lickschat P.; Weißmantel S. High-Quality Surface Treatment Using GHz Burst Mode with Tunable Ultrashort Pulses. Appl. Surf. Sci. 2020, 531, 147270 10.1016/j.apsusc.2020.147270.

[ref31] Wlodarczyk K. L.; Schille J.; Naumann L.; Lopes A. A.; Bitharas I.; Bidare P.; Dondieu S. D.; Blair P.; Loeschner U.; Moore A. J.; Mercedes Maroto-Valer M.; Hand D. P. Investigation of an Interlaced Laser Beam Scanning Method for Ultrashort Pulse Laser Micromachining Applications. J. Mater. Process. Technol. 2020, 285, 116807 10.1016/j.jmatprotec.2020.116807.

[ref32] Neuenschwander B.; Kramer T.; Lauer B.; Jaeggi B. Burst Mode with Ps- and Fs-Pulses: Influence on the Removal Rate, Surface Quality, and Heat Accumulation. Laser Applications in Microelectronic and Optoelectronic Manufacturing (LAMOM) XX 2015, 9350, 93500U 10.2351/1.5096083.

[ref33] Neuenschwander B.; Jaeggi B.; Schmid M. From Fs to Sub-Ns: Dependence of the Material Removal Rate on the Pulse Duration for Metals. Phys. Procedia 2013, 41, 794–801. 10.1016/j.phpro.2013.03.150.

[ref34] Jia X.; Zhao X. Numerical Study of Material Decomposition in Ultrafast Laser Interaction with Metals. Appl. Surf. Sci. 2019, 463, 781–790. 10.1016/j.apsusc.2018.08.225.

[ref35] Lopez J.; Niane S.; Bonamis G.; Balage P.; Audouard E.; Hönninger C.; Mottay E.; Manek-Hönninger I. Percussion Drilling in Glasses and Process Dynamics with Femtosecond Laser GHz-Bursts. Opt. Express 2022, 30 (8), 12533–12544. 10.1364/OE.455553. 35472887

[ref36] Zhao X.; Shin Y. C. Laser-Plasma Interaction and Plasma Enhancement by Ultrashort Double-Pulse Ablation. Appl. Phys. B: Laser Opt. 2015, 120 (1), 81–87. 10.1007/s00340-015-6102-4.

[ref37] Povarnitsyn M. E.; Levashov P. R.; Knyazev D. V. Simulation of Ultrafast Bursts of Subpicosecond Pulses: In Pursuit of Efficiency. Appl. Phys. Lett. 2018, 112 (5), 051603 10.1063/1.5012758.

[ref38] Förster D. J.; Faas S.; Gröninger S.; Bauer F.; Michalowski A.; Weber R.; Graf T. Shielding Effects and Re-Deposition of Material during Processing of Metals with Bursts of Ultra-Short Laser Pulses. Appl. Surf. Sci. 2018, 440, 926–931. 10.1016/j.apsusc.2018.01.297.

[ref39] Hirsiger T.; Gafner M.; Remund S. M.; Chaja M. W.; Urniezius A.; Butkus S.; Neuenschwander B. Machining Metals and Silicon with GHz Bursts: Surprising Tremendous Reduction of the Specific Removal Rate for Surface Texturing Applications. In Laser Applications in Microelectronic and Optoelectronic Manufacturing (LAMOM) XXV; SPIE, 2020; Vol. 11267, p 112670T.

[ref40] Kam D. H.; Bhattacharya S.; Mazumder J. Control of the Wetting Properties of an AISI 316L Stainless Steel Surface by Femtosecond Laser-Induced Surface Modification. J. Micromechanics Microengineering 2012, 22 (10), 105019 10.1088/0960-1317/22/10/105019.

[ref41] Peng E.; Bell R.; Zuhlke C. A.; Wang M.; Alexander D. R.; Gogos G.; Shield J. E. Growth Mechanisms of Multiscale, Mound-like Surface Structures on Titanium by Femtosecond Laser Processing. J. Appl. Phys. 2017, 122 (13), 133108 10.1063/1.4990709. 30410187 PMC6218944

[ref42] Lickschat P.; Metzner D.; Weißmantel S. Manufacturing of High Quality 3D Microstructures in Stainless Steel with Ultrashort Laser Pulses Using Different Burst Modes. J. Laser Appl. 2021, 33 (4), 042002 10.2351/7.0000437.

[ref43] Lickschat P.; Metzner D.; Weißmantel S. Burst Mode Ablation of Stainless Steel with Tunable Ultrashort Laser Pulses. J. Laser Appl. 2021, 33 (2), 022005 10.2351/7.0000271.

[ref44] Jaeggi B.; Remund S.; Zhang Y.; Kramer T.; Neuenschwander B. Optimizing the Specific Removal Rate with the Burst Mode under Varying Conditions. J. Laser Micro Nanoeng. 2017, 12 (3), 258–266.

[ref45] Kramer T.; Zhang Y.; Remund S.; Jaeggi B.; Michalowski A.; Grad L.; Neuenschwander B. Increasing the Specific Removal Rate for Ultra Short Pulsed Laser-Micromachining by Using Pulse Bursts. J. Laser Micro Nanoeng. 2017, 12 (2), 107–114. 10.2961/jlmn.2017.02.0011.

[ref46] Metzner D.; Lickschat P.; Weißmantel S. Optimization of the Ablation Process Using Ultrashort Pulsed Laser Radiation in Different Burst Modes. J. Laser Appl. 2021, 33 (1), 012057 10.2351/7.0000352.

[ref47] Tsibidis G. D.; Fotakis C.; Stratakis E. From Ripples to Spikes: A Hydrodynamical Mechanism to Interpret Femtosecond Laser-Induced Self-Assembled Structures. Phys. Rev. B 2015, 92 (4), 041405 10.1103/PhysRevB.92.041405.

