# Supplementary material for: Ultrasmooth Micromilling of Stainless Steel by Ultrashort Pulsed Laser Ablation Using MHz Bursts
Source: ACS Appl Mater Interfaces. 2025 Jan 22;17(5):7989–8002. doi: 10.1021/acsami.4c19517 (PMC11803565; doi:10.1021/acsami.4c19517)
Supplement: Supplementary file 2 — am4c19517_si_002.pdf [file am4c19517_si_002.pdf]

Supporting Information

Ultrasmooth micro-milling of stainless steel by ultrashort pulsed laser ablation using MHz bursts

Xiao Jia [a,*], Folkert Vrijburg [b], Wei Zhang [a], Max Groenendijk [c], Yutao Pei [a,*]

[a] Department of Advanced Production Engineering, Engineering and Technology Institute Groningen, Faculty of Science and Engineering, University of Groningen, Nijenborgh 4, Groningen, 9747 AG, the Netherlands

[b] Philips Personal Care, Oliemolenstraat 5, Drachten, 9203 ZN, the Netherlands

[c] Lightmotif B.V., Pantheon 12, Enschede, 7521 PR, the Netherlands

S1 Numerical simulation

In this model, a Gaussian-shape laser beam propagates along the +*z* direction, perpendicular to the material surface, while the laser beam scans in the +x direction, parallel to the material surface. The initial material surface is located at z=0. The region of interest (the black circle in Fig. 2), with a size of the laser focal spot, is placed at the center of the material surface (denoted as O). During laser scanning, the material within this region is heated by laser pulse irradiation, followed by subsequent cooling as the laser beam passes through this region. Due to overlapping of laser pulses, this region is repetitively irradiated by multiple pulses. A scan length of $4\omega_{0,}$ starting from a distance of $2\omega_0$ away from the center O (on the left-hand side), is considered in the simulation to capture the thermodynamic behavior within the region of interest. Meanwhile, this scan length is long enough to ensure that the ablation crater and melting layer are fully developed and their dimensions can be properly determined. It is worthwhile to mention that the region of interest can represent any position on the material surface and the simulation can reflect the dominating thermodynamic process throughout the USP laser burst ablation.

* Corresponding author. Email address: xiao.jia@rug.nl (X. Jia), y.pei@rug.nl (Y. Pei)

The material undergoes heating by absorbing the laser pulse energy and the temperature increase following single-pulse irradiation can be described as follows[1]

$$\Delta T(t,x,z) = \Delta T_0 \cdot \frac{\omega_0^2}{\omega_0^2+4Dt} \cdot \left(\frac{l_z^2}{l_z^2+4Dt}\right)^{1/2} \exp\left(-\frac{x^2}{\omega_0^2+4Dt} - \frac{z^2}{l_z^2+4Dt}\right) \quad \text{(S1)}$$

where ΔT_0 is the maximum temperature increase resulted from a single pulse irradiation, ω_0 is the focal spot radius, D is the thermal diffusivity, and l_z is the thermal penetration depth[2]. ΔT_0, D and l_z are calculated as follows, $\Delta T_0 = \frac{F_p \cdot (1-R)}{l_z \cdot C_p \cdot \rho}$, $D = \frac{\kappa}{C_p \cdot \rho}$ and $l_z = a(m_{ion}/3m_e)^{1/2}$, where F_p is the laser pulse peak fluence, R is the surface reflectivity, C_p is the heat capacity, ρ is the mass density, κ is the thermal conductivity, a is the average interatomic distance, m_{ion} and m_e are the mass of the ion and the electron, respectively. Taking a as 2.87 Å[3], l_z is calculated as 52.8 nm, which is in good agreement with the estimation by Sedao *et al.*[4]

Based on Eq. (S1), the temperature distribution after laser bursts irradiation is expressed as

$$T(t,x,z) = \sum_{n=0}^{N_b-1} \sum_{m=0}^{N_p-1} \Delta T\left[\left(t - \tau_{n,m}\right), \left(x - \tau_{n,m} \cdot v_s\right), z\right] + T_r \quad \text{(S2)}$$

where N_b is the number of bursts within the scan length, N_p is the number of pulses within each burst, $\tau_{n,m}$ is the time interval between adjacent laser pulses, and v_s is the laser scanning speed. $\tau_{n,m}$ and v_s are calculated as $\tau_{n,m} = n\Delta\tau_b + m\Delta\tau_p$ and $v_s = \frac{2\omega_0 \cdot f_b}{1-\varphi}$, where $\Delta\tau_b$ is the separation time between bursts, calculated as $\Delta\tau_b = \frac{1}{f_b}$, $\Delta\tau_p$ is the separation time between pulses within a burst, calculated as $\Delta\tau_p = \frac{1}{f_p}$, f_b and f_p are the inter-burst and intra-burst repetition rates, respectively, φ is the overlap ratio between laser bursts, and T_r is the room temperature taken as 300 K.

The simulation domain length (in x direction) is 100 μm to accommodate the scan length ($4\omega_0$), and the domain width (in z direction) is initially set as 10 μm and able to self-extended to be greater than the melting depth. The ablation and melting depths are determined based on the boiling point (T_b) and melting point (T_m) of stainless steel, respectively, in temperature distribution predicted by Eq. (S2). The physical properties of stainless steel used in the simulation are listed in Table S1.

Laser parameters settings (intra-burst repetition rate, inter-burst repetition rate, pulse fluence, PpB and burst overlap ratio) used in the simulation are the same as the experiments employed.

Table S1 Physical properties of stainless steel [2,5,6]

Heat capacity, C_p [J/(kg·K)]	510
Density, ρ [kg/m^3]	7930
Thermal conductivity, κ [W/(m·K)]	16.0
Reflectivity, R	0.85
Thermal penetration depth, l_z [nm]	52.8
Boiling temperature, T_b [K]	3134
Melting temperature, T_m [K]	1683

References

(1) Shimizu, M.; Sakakura, M.; Ohnishi, M.; Shimotsuma, Y.; Nakaya, T.; Miura, K.; Hirao, K. Mechanism of Heat-Modification inside a Glass after Irradiation with High-Repetition Rate Femtosecond Laser Pulses. *J. Appl. Phys.* **2010**, *108* (7), 073533.

(2) Nolte, S.; Momma, C.; Jacobs, H.; Tünnermann, A.; Chichkov, B. N.; Wellegehausen, B.; Welling, H. Ablation of Metals by Ultrashort Laser Pulses. *J. Opt. Soc. Am. B* **1997**, *14* (10), 2716–2722.

(3) *https://periodictable.com/Elements/026/data.html*.

(4) Sedao, X.; Lenci, M.; Rudenko, A.; Faure, N.; Pascale-Hamri, A.; Colombier, J. P.; Mauclair, C. Influence of Pulse Repetition Rate on Morphology and Material Removal Rate of Ultrafast Laser Ablated Metallic Surfaces. *Opt. Lasers Eng.* **2019**, *116*, 68–74.

(5) *https://www.azom.com/properties.aspx?ArticleID=960*.

(6) Haynes, W. *CRC Handbook of Chemistry and Physics*; CRC Press: Boca Raton, 2014.
